# Expanding the LuxR-type receptor functional repertoire: Protein-protein interactions in quorum sensing regulation

**DOI:** 10.1371/journal.ppat.1014091

**Published:** 2026-04-01

**Authors:** Caleb P. Mallery, Jon E. Paczkowski

**Affiliations:** 1 Department of Biomedical Sciences, University at Albany, College of Integrated Health Sciences, Albany, New York, United States of America; 2 Division of Genetics, Wadsworth Center, New York State Department of Health, Albany, New York, United States of America; 3 The RNA Institute, University at Albany, College of Arts and Sciences, Albany, New York, United States of America; Monash University, Australia

## Abstract

Quorum sensing (QS) enables bacteria to coordinate gene expression in response to population density, with LuxR-type transcription factors playing a central role in this process for many Gram-negative species. Traditionally understood as ligand-activated transcriptional regulators, LuxR-type proteins are increasingly recognized as targets of diverse protein-protein interactions (PPIs) that modulate their activity, stability, and specificity. This review synthesizes emerging insights into the regulatory landscape of LuxR-type receptors, focusing on direct PPIs that expand the functional repertoire of LuxR-type receptors. We classify LuxR-interacting partners into negative regulators, dual or context-dependent regulators, and global regulators, highlighting the well-characterized system of PqsE-RhlR in *Pseudomonas aeruginosa* as well as emerging candidates. We discuss how these interactions influence LuxR-type receptor outputs beyond canonical ligand-mediated gene regulation, through stabilization of active conformations, inhibition of dimerization, proteolytic regulation, and potential recruitment of transcriptional machinery. By examining functional parallels, structural determinants, and environmental integration, we highlight a framework in which LuxR-type receptors are subject to diverse regulatory PPI that allow for the fine-tuning of QS output. Understanding these regulatory mechanisms offers promising alternatives to conventional QS disruption strategies, for targeted interference with bacterial virulence and communication.

## Introduction

**Fig 1:**
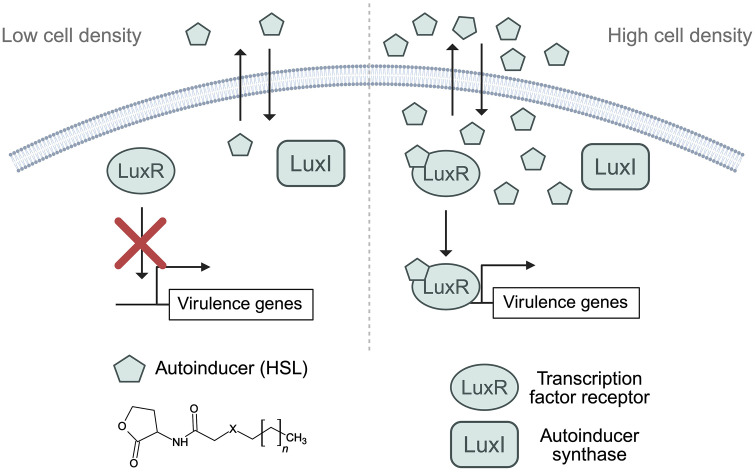
Canonical LuxIR-type quorum sensing (QS) system in Gram-negative bacteria. At low cell density (LCD; left panel), LuxR, a LuxR-type transcription factor (light-green ovals), remains inactive due to the absence of its cognate autoinducer, an acyl-homoserine lactone (AHL) signal (light-green pentagons). LuxI, the autoinducer synthase (light-green squares), constitutively produces AHLs at basal levels that accumulate in the extracellular environment. At LCD, AHL concentration is insufficient to activate LuxR or trigger a population-level response. At high cell density (HCD; right panel), AHL levels are elevated and diffuse back into cells, enabling binding to LuxR, which promotes receptor folding and dimerization, DNA binding, and activation of LuxR-dependent genes, including those involved in virulence. Activated LuxR promotes transcription of its own gene and *luxI*, establishing a positive feedback loop that amplifies QS. For simplicity, LuxR is depicted as a monomer, though it functions as a dimer when bound to AHL. The red “X” in the LCD panel indicates that LuxR is inactive and unable to bind DNA or activate transcription in the absence of sufficient AHL. The chemical structure of a basic AHL is shown in black. All AHLs contain a homoserine lactone ring and an acyl side chain that varies in length (n), oxidation state, and the presence of substituents (X). Created in BioRender. Paczkowski, J. (2026) https://BioRender.com/bzj00cg.

Quorum sensing (QS) is a widespread form of bacterial communication that enables cells to coordinate gene expression in response to population density [1–3]. In Gram-negative bacteria, QS is often mediated by acyl-homoserine lactones (AHLs), which are small molecules that freely diffuse across membranes and accumulate in the extracellular environment as the population increases [4–6]. AHLs are synthesized by LuxI enzymes, which function in tandem with their cognate LuxR-type receptors to form a tightly regulated positive feedback loop: activated LuxR promotes transcription of both QS-regulated target genes and the *luxI* synthase gene, thereby amplifying ligand production as population density increases (Fig 1) [2,3,7,8]. Once a critical threshold is reached, AHLs bind in the ligand-binding pocket (LBP) of the LuxR-type receptor to induce conformational changes that promote dimerization and DNA binding, thereby activating or repressing QS-regulated genes [9–11]. LuxR-type receptors are broadly classified into three distinct classes: 1) canonical AHL-activated receptors [12]; 2) receptors that function in the absence of AHL [13–15]; and 3) “solo” LuxR-type proteins that lack a cognate AHL synthase and may sense alternative ligands or those produced by other QS-pathways [16–18]. Additional subclasses have been described based on atypical ligand-binding profiles, a degree of ligand selectivity [19], or evolutionarily derived functions, such as transcriptional anti-activation or ligand-independent dimerization [13,20–22].

**Fig 2:**
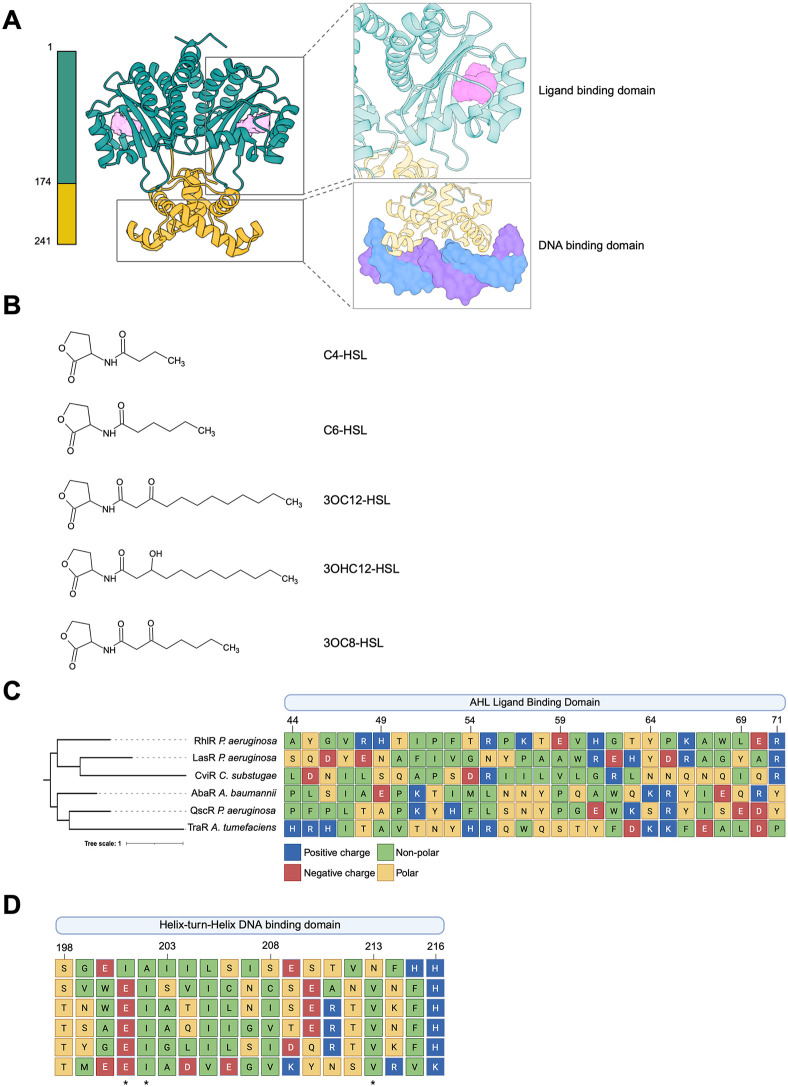
Conserved structural and sequence features of LuxR-type quorum sensing receptors. **(A)** Structural model of *P. aeruginosa* RhlR, highlighting the modular architecture that defines LuxR-type receptors: an N-terminal acyl-homoserine lactone (AHL) ligand-binding domain (LBD; turquoise) and a C-terminal helix-turn-helix (HTH) DNA-binding domain (DBD; yellow). The solvent-accessible volume of the LBD ligand-binding pocket is shown in light pink to provide structural context. Structures are derived from PDB IDs 8DQ0 and 8DQ1 [82], and all structure images were generated using ChimeraX. **(B)** Panel of AHL signal molecules recognized by the cognate receptors as in panel C. C4-HSL; *P. aeruginosa*; RhlR, C6-HSL; *C. subtsugae*; CviR, 3OC8-HSL; *A. tumefaciens*; TraR, 3OC12-HSL; LasR; 3OHC12-HSL; *A. baumannii*; AbaR. **(C)** Multiple sequence alignment (MSA) of LuxR-type receptors discussed in this review, spanning residues 44–72, which correspond to a conserved portion of the LBD. Amino acids are colored by physicochemical properties (positive, negative, polar, and non-polar, as indicated). A maximum-likelihood phylogenetic tree based on full-length sequences is shown to the left of the alignment. **(D)** Sequence alignment of the HTH DNA binding domains (residues 198–217) from the same set of receptors shown in panel B. The phylogenetic tree is omitted for clarity. Conserved residues within the HTH motif are denoted with an asterisk.

LuxR-type receptors regulate diverse biological processes, including virulence factor production, biofilm formation, secondary metabolite production, and motility [23]. The receptors are defined by a modular architecture (Fig 2A): a variable N-terminal ligand-binding domain (LBD) that confers AHL ligand specificity (Fig 2B and 2C), and a conserved C-terminal helix-turn-helix (HTH) domain responsible for DNA recognition (Fig 2D) [24]. In the canonical model, AHL-binding stabilizes the receptor, enabling dimerization, and subsequent binding to conserved promoter motifs (often termed “Lux boxes”) to modulate transcription [25,26]. While this model has provided a foundational understanding of LuxR-type receptor activation, recent work has uncovered an additional layer of regulation where receptor function is modulated by protein-protein interactions (PPIs). Over the past decades, a growing number of protein-binding partners have been identified that interact directly with LuxR-type receptors to modulate their activity [27–34], expanding the regulatory potential of QS circuits beyond ligand-binding alone.

**Fig 3:**
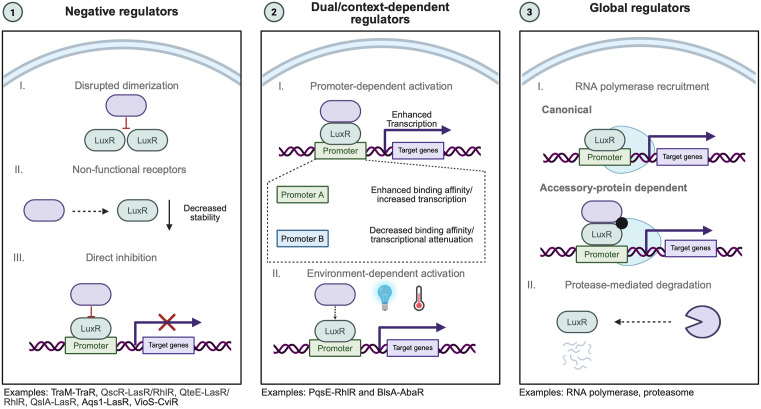
LuxR-type receptors are subject to diverse regulatory protein-protein interaction mechanisms that influence their activity as transcriptional regulators. LuxR-type QS receptors (light-green ovals) are subject to regulation by a range of protein interaction partners (purple ovals), which modulate receptor function through distinct mechanisms. Negative regulators inhibit LuxR-type receptor function by disrupting dimerization, decreasing receptor stability, and inhibiting DNA binding. Representative examples include TraM-TraR (*A. tumefaciens*)*,* QscR-LasR/RhlR QteE-LasR/RhlR, Aqs1-LasR (all *P.s aeruginosa*), and VioS-CviR (*C. subtsugae*). Dual or context-dependent regulators modify LuxR-type receptor activity in a promoter-, ligand-, or environmental-dependent manner. Examples include PqsE-RhlR (*P. aeruginosa*) and BlsA-AbaR (*A. baumannii*), which increase DNA binding affinity and/or transcriptional output under specific conditions. Global regulators modulate LuxR-type receptor function through transcriptional engagement or post-translational control, including RNA polymerase-mediated activation and protease-dependent receptor degradation. Created in BioRender. Paczkowski, J. (2026) https://BioRender.com/akj8r1w.

This review synthesizes current knowledge of LuxR-type regulators across Gram-negative bacteria that influence QS through direct or indirect PPI. For clarity, we categorize these LuxR interactors according to their overall functional effects on LuxR activity: negative regulators, which directly inhibit LuxR folding, stability, or activity; dual or context-dependent regulators, whose effects vary across promoters or environmental conditions; and global regulators, which integrate receptor function into broader physiological networks (Fig 3). We highlight both well-characterized examples and emerging candidates of LuxR-type regulators to illustrate the complexity and versatility of LuxR-type receptor regulation, underscoring the central role of PPIs in tuning bacterial gene regulation and communication.

### Why do LuxR-type proteins require accessory proteins?

LuxR-type transcription factors frequently rely on PPIs to regulate receptor availability, stability, and transcriptional output. Although these receptors are classically described as AHL-responsive regulators, many LuxR-type proteins possess LBD that are unstable, aggregation-prone, or only partially functional in isolation. Accessory proteins can buffer these biophysical constraints by stabilizing specific receptor conformations, modulating dimerization, or promoting productive DNA engagement. In other cases, PPIs act in the opposite direction, restraining receptor activity to prevent premature or inappropriate activation of QS programs. Beyond receptor stabilization, PPIs provide a mechanism to control the timing, specificity, and amplitude of LuxR-dependent transcription. Negative regulators can delay activation at low-cell density (LCD), suppress self-sensing, accelerate QS shut-off following signal dilution or dispersal, or serve as nodes for integrating regulatory inputs from other systems, allowing cues beyond AHL concentration alone to permit QS activation. Global regulators can couple LuxR activity to broader physiological processes, including proteolysis and transcriptional machinery availability. Together, these interactions ensure that QS outputs reflect not only signal accumulation but also cellular state and environmental context.

PPI-based regulation is a common strategy in bacterial transcriptional regulation. Throughout bacterial regulatory networks, transcription factors are frequently controlled by non-DNA binding partner proteins, including anti-sigma factors [35], NusA/NusG-mediated control of transcriptional elongation [36], AraC-family co-regulators [37], and phosphorylation-dependent two-component systems [38]. LuxR-associated accessory proteins appear to operate under similar principles, enabling QS circuits to integrate diverse inputs without altering core receptor architecture. A growing body of evidence demonstrates that LuxR-type receptors can be modulated through direct PPIs that either enhance or inhibit receptor activity, alter promoter specificity, or redirect transcriptional outcomes. Notably, these accessory proteins generally do not bind DNA themselves, distinguishing them from canonical transcriptional activators such as sigma factors or CRP (cAMP receptor protein), which act primarily at the DNA-RNA polymerase (RNAP) interface [39]. Instead, LuxR accessory proteins act directly on the receptor, shaping its functional state before or during promoter engagement, or by influencing receptor stability and turnover.

Why some LuxR-type receptors require post-translational regulation while others appear largely ligand sufficient remains an open question. Current evidence suggests that accessory proteins compensate for intrinsic receptor instability, suppress noise and self-sensing, enforce appropriate activation thresholds, or couple QS outputs to environmental and physiological cues that are not encoded by AHL concentration alone. The systems reviewed here illustrate how LuxR-type receptor activity can be fine-tuned by diverse PPI modes of regulation rather than AHL-binding alone.

## Negative regulation of LuxR-type receptors by protein-protein interaction

QS is often described as a population-level signaling mechanism: bacteria produce, release, and collectively detect diffusible AHL to synchronize gene expression once a critical concentration is reached. However, theoretical models and experimental observations suggest that under certain conditions, QS circuits can also support self-sensing [40–43]. In this model, individual cells may activate QS-regulated genes independently of population density, potentially leading to heterogeneous or premature gene expression. To constrain this potential instability, many QS systems encode negative regulators that restrain LuxR activity post-translationally. Negative regulators act through PPIs to inhibit LuxR function even in the presence of AHL. Although individual regulators employ distinct strategies, including receptor sequestration, inhibition of dimerization, and destabilization, the defining feature of this class is their net inhibitory effect on LuxR-dependent transcription. Importantly, distinctions such as “anti-activation” versus “repression” are largely functional rather than mechanistic; LuxR-interacting negative regulators instead span a continuum of inhibitory effects, ranging from delayed activation to sustained suppression of transcriptional output. In this section, we review known examples of negative regulators of LuxR-type receptors by direct PPI (Fig 3).

### TraM establishes a paradigm for PPI-mediated LuxR inhibition

The TraR-TraM system of *Agrobacterium tumefaciens* provides a foundational example of LuxR negative regulation via direct PPI. In *A. tumefaciens*, QS regulates the conjugative transfer of the Ti plasmid, which carries virulence genes required for plant infection and is a metabolically costly process requiring tight temporal control [34,44,45]. This system is governed by a canonical LuxIR-type system: TraI synthesizes the AHL 3-oxo-octanoyl homoserine lactone (3OC8-HSL), which binds to and activates the LuxR-type transcription factor TraR and stabilizes it [1,34]. Ligand-bound TraR dimerizes and activates transcription of genes required for plasmid replication and transfer.

**Fig 4:**
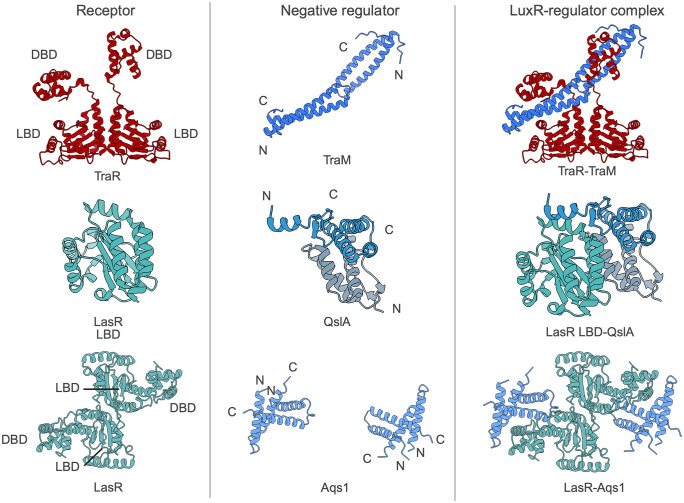
Structural basis of anti-activation in LuxR-type quorum sensing receptors. **(A)** Structure of TraM-TraR complex from homologous *Rhizobium* sp. strain NGR234 proteins (PDB ID: 2Q0O [49]). Shown sequentially are: TraR homodimer alone (red), TraR homodimer (blue), and the full 2:2 TraM-TraR complex. TraM functions as an anti-activator by binding to TraR, inducing an allosteric conformational change in the protein and preventing DNA binding. **(B)** Structure of the QslA-LasR ligand-binding domain (LBD) complex from *Pseudomonas aeruginosa* (PDB ID: 4NG2 [61]). The complex consists of 2:1 stoichiometry, with a QslA homodimer (blue and gray) interacting with a single monomer of the LasR LBD (cyan), obstructing dimer formation and preventing LasR activation. **(C)** Structure of Aqs1-LasR complex from *P. aeruginosa* (PDB ID: 6V7W [30]). The complex consists of an Aqs1 dimer which binds to the DNA-binding domain of a LasR monomer preventing DNA binding. These co-crystal structures illustrate distinct anti-activation mechanisms: disruption of DNA binding (TraM-TraR and Aqs1-LasR), and disruption of receptor dimerization (QslA-LasR). All structure images were generated using ChimeraX.

Initial observations suggested that TraM functions as a negative regulator of TraR, as overexpression of *traM* suppressed the activation of TraR-dependent genes [46]. To determine whether this inhibition was due to direct interaction, yeast two-hybrid (Y2H) assays demonstrated that TraM and TraR physically interact *in vivo* [46]. Subsequent *in vitro* binding assays and mutational analysis confirmed that the interaction involves the C-terminal DNA binding domain (DBD) of TraR [47,48]. Structural studies provided further insight into this mechanism. A crystal structure of the TraM-TraR complex revealed that TraM forms a heterotetrametric complex with TraR, binding at a surface distal to the DBD (Fig 4; top panel) [49]. This interaction induces a conformational change that allosterically prevents DNA binding and enables stepwise dissociation of TraR from DNA [49]. Functionally, this interaction prevents TraR DNA binding and, therefore, its ability to function as a transcriptional activator. As a result, TraM binding to TraR effectively blocks transcription of target genes, even in the presence of AHL.

Later work showed that the TraM mechanism is much more complex than initially appreciated. In the *A. tumefaciens* strain A6, a second homologous protein, TraM2 was discovered [50]. Although encoded at a distinct genomic locus, TraM2 also interacts with TraR and contributes to the repression of Ti plasmid conjugation, suggesting a redundant or fine-tuning role in QS regulation. Additionally, a truncated TraR-like protein encoded by *A. tumefaciens* octopine-type strains, TrlR, which lacks the C-terminal DNA binding domain, was found to bind to and form inactive heterodimers with TraR, further inhibiting its activity [48,51]. These findings highlight a sophisticated network of anti-activation that operates at multiple levels: direct sequestration (TraM and TraM2) and dominant-negative inhibition (TrlR), to ensure tight control over the energetically expensive process of plasmid transfer. This system set a precedent for how LuxR-type receptors can be negatively regulated by PPI, a theme that occurs in diverse QS systems across Gram-negative bacteria.

### Negative regulators of LuxR-type receptors in *Pseudomonas* *aeruginosa*

QS in *P. aeruginosa* is subject to extensive post-translational regulation, with multiple proteins acting to restrain the LuxR-type receptors LasR and RhlR and prevent premature activation of virulence-associated genes. These negative regulators operate through distinct and, in some cases, partially overlapping post-translational strategies to collectively suppress self-sensing at LCD through direct and indirect modulation of receptor activity.

#### QscR functions as a checkpoint for QS activation.

QscR is an orphan LuxR-type receptor in *P. aeruginosa* that fine-tunes QS activation through two distinct mechanisms: direct PPI with other receptors and transcriptional control of an anti-activation operon. Genetic screens first revealed that *qscR* mutants exhibit premature and enhanced activation of QS-regulated genes, implicating QscR in delaying QS activation [52]. Mechanistically, QscR has been shown to interact directly with LasR and RhlR in the absence of AHL, as demonstrated by fluorescence anisotropy and *in vivo* cross-linking experiments that detected heterodimer formation between these receptors [32]. Notably, these interaction studies were performed using *qscR* overexpression strains. While these data support the capacity of QscR to interact with LasR and RhlR, the extent to which these interactions occur under physiological conditions remains an important consideration.

In parallel, QscR directly activates transcription of the PA1895-1897 operon (PAO1 gene numbering), whose gene products function to delay QS induction through an unresolved mechanism. Notably, deletion of PA1895-1897 phenocopies the *qscR* mutant, suggesting that the anti-activation phenotype of QscR is likely mediated, at least in part, through this operon [53]. Because both QscR-dependent transcriptional activation and QscR-LuxR interaction have primarily been characterized in overexpression systems, it remains possible that the gene products of the QscR regulon contribute substantially to QS restraint, either independently or in concert with direct QscR-LuxR interactions.

QscR binds a broad range of AHLs [19,54], but regulates a narrow regulon of genes [53]. This disconnect between broad ligand sensitivity and limited transcriptional regulation suggests that the primary role of QscR is not to regulate a complex gene network but to act as a regulatory “brake” early in QS induction, restraining QS activation until higher signal thresholds are reached [53]. Together, these findings support a model in which QscR functions as a QS checkpoint, restraining the timing and amplitude of QS induction via both physical interference with key receptors and the transcriptional output of an anti-activation operon. The role of environmental conditions, growth state, or interspecies signals in influencing QscR abundance, heterodimer formation, and expression of the PA1895–1897 operon are compelling open questions.

#### QteE: an anti-activator that destabilizes LasR and RhlR through an unknown mechanism.

QteE is a second *P. aeruginosa* anti-activator, identified in a screen for regulators that prevent premature QS activation [55]. Expression of *qteE* represses multiple QS-regulated virulence factors, including rhamnolipids, elastase, and pyocyanin, even in the presence of saturating exogenous AHLs [55]. Since AHL levels in the medium remained unaffected, this suggested that QteE does not act by limiting signal production or degradation, but rather QteE might directly interfere with receptor function. Subsequent experiments showed that QteE reduces protein stability of both LasR and RhlR without altering their transcription or translation [55]. Timed expression experiments demonstrated that QteE decreased LasR levels in which *qteE* expression was induced either before or after LasR accumulation, including conditions where LasR was already present and stabilized by exogenous AHL. Pulse-chase assays further confirmed that QteE accelerates LasR degradation without altering its synthesis. These findings support a model in which QteE targets receptors for degradation, potentially by forming inhibitory complexes, although the precise stoichiometry and physical nature of these complexes remains undefined. Notably, although a direct interaction between QteE and LasR or RhlR has been proposed, it has not yet been experimentally demonstrated.The ability of QteE to destabilize LuxR-type receptors likely underlies its broader effects on QS output and virulence factor expression. To this end, overexpression of *qteE* suppressed expression of QS targets such as *lasB*, *rhlA*, *phzA1*, as well as attenuating virulence in fruit fly and potato infection models [56]. Transcriptomic studies revealed that QteE acts additively with QscR and a third QS negative regulator, QslA, to limit the pool of soluble LasR and modulate the dynamics of QS activation [57]. Loss of *qteE* leads to premature and elevated QS activation in *P. aeruginosa* transcriptional reporter strains [55]; however, this heightened activity does not enhance pathogenicity in murine chronic lung infection models, highlighting the context-dependence of QS regulation [58]. Whether QteE functions through direct receptor binding or by modulating proteolytic pathways remains a key open question.

#### QslA disrupts LasR homodimerization.

LasR is a key LuxR-type QS receptor in *P. aeruginosa*, activating the expression of virulence genes in response to its cognate AHL, 3-oxo-C12-homoserine lactone (3OC12-HSL) [59]. Ligand-binding promotes LasR homodimerization, a conformation required for DNA binding and transcriptional activation [11,60]. QslA was identified in a transposon mutagenesis screen as a negative regulator of LasR activity [33]. Disruption of *qslA* led to increased LasR-dependent gene expression, prompting researchers to hypothesize that QslA might act as an inhibitor of LasR function through a physical interaction. Biochemical studies confirmed that QslA directly binds to LasR to form a nonfunctional heterodimer, preventing LasR homodimerization thereby preventing downstream gene expression of the LasR regulon. Structural and mutagenesis studies mapped the interaction interface to the LasR LBD, identifying key residues required for QslA-mediated inhibition [61] (Fig 4; middle panel). This mechanism, blocking receptor dimerization through competitive binding, represents a third strategy of anti-activation distinct from QscR sequestration and QteE-mediated degradation. Interestingly, *qslA* is expressed in the laboratory strain PAO1 but is absent in the hypervirulent UCBPP-PA14 strain (PA14), a difference that may contribute to elevated QS activity and virulence in PA14 under laboratory conditions [62]. Whether this strain-specific pattern of *qslA* expression is conserved across other *P. aeruginosa* isolates remains to be determined. More recently, QteE and QslA were shown to prevent self-sensing at LCD; in a *qteE qslA* double mutant, cells prematurely activated QS independently of population-wide signal accumulation, producing a bimodal expression pattern across the population [43]. These results support a broader model in which anti-activators help preserve QS as a synchronized, population-level behavior.

#### Aqs1 is a phage-encoded negative regulator of LasR.

Distinct from the chromosomally encoded negative regulators described above, QS in *P. aeruginosa* can also be attenuated by a temperate phage-encoded protein. Aqs1 was identified as a phage-encoded factor that suppresses multiple QS-regulated phenotypes, including pyocyanin (PYO) production, swarming motility, and protease and rhamnolipid production [30]. Constitutive expression of *aqs1* produced a phenotype closely resembling a *lasR* mutant, including changes in PYO production, autolysis, and virulence factor production, suggesting negative regulation of LasR by Aqs1. Additionally, Aqs1 is rapidly expressed following initiation of phage infection. Notably, deletion of *aqs1* from the phage genome increased production of the PQS-signaling molecule, in agreement with the proposed negative regulation of LasR, a canonical positive regulator of the PQS system. Biochemical and genetic analyses support a model in which Aqs1 directly interacts with LasR to inhibit QS output. Aqs1 interaction with LasR was first observed in bacterial adenylate cyclase two-hybrid (BACTH assays). This interaction was confirmed by the X-ray crystal structure of full-length LasR bound to Aqs1; notably, this was the first full-length structure of LasR. The structure revealed that Aqs1 binds to the DBD of LasR, suggesting that Aqs1 inhibits LasR activity by direct PPI inhibiting LasR DNA binding (Fig 4; bottom panel). In support of this model, LasR co-purified with Aqs1 was unable to bind the *lasB* operator sequence in electrophoretic mobility shift assays (EMSA). The structure of LasR-Aqs1 allowed for the identification of Aqs1 residues that are necessary for negative regulation of QS-phenotypes, which is shared among several Aqs1 homologs, suggesting that this mechanism may be conserved in other bacteriophages. Therefore, Aqs1 may serve as a single protein that effectively silences a suite of anti-phage defenses by inhibition of QS-pathways. The prophage origin of Aqs1 further highlights the capacity of horizontally acquired elements to directly interface with host QS circuits, providing an additional layer of regulatory plasticity. Whether Aqs1 expression is dynamically regulated during infection or in response to environmental cues, and how this regulation intersects with host fitness and phage biology, remain important open questions.

### Negative regulation of CviR by VioS – an emerging candidate

Repression of bacterial QS is most often achieved indirectly, via transcriptional regulators that silence QS synthase or receptor genes [63]. Well-characterized examples include the DNA-binding repressor RsaL [64] in *P. aeruginosa* and the Gac two-component system in various Gram-negative species [65,66]. By comparison, direct inhibition of a LuxR-type receptors by PPI, to our knowledge, has been described in relatively few systems. One such example is found in *Chromobacterium* species, where the protein VioS negatively regulates the LuxR-type receptor CviR [31,67]. In *Chromobacterium subtsugae* (formerly *C. violaceum* ATCC 31532), CviR activates violacein production and other virulence-associated genes in response to its cognate AHL [68]. VioS was identified through follow-up analysis of *vitR*, a TetR-family transcriptional repressor identified in a transposon mutagenesis screen for altered siderophore production [31]. Transcriptome analysis of a ∆*vitR* mutant revealed *vioS* as one of the most highly upregulated genes at high cell density (HCD), placing VioS downstream of VitR in a multilayered regulatory cascade. Genetic and heterologous reporter assays indicated that VioS suppressed CviR-dependent transcription post-translationally. Overexpression of *vioS* attenuates CviR-dependent phenotypes, including violacein and protease production, without altering *cviR* transcription or AHL levels [31]. In a heterologous *Escherichia coli* reporter system, co-expression of VioS reduced activation of a *vioA* promoter-*lacZ* fusion by CviR more than sixfold, even in the presence of *N*-hexanoyl-L-homoserine lactone (C6-HSL), demonstrating that VioS is sufficient to repress CviR-mediated activation at the *vioA* promoter [67]. Importantly, this repressive effect was specific to the CviR system, as VioS did not repress CepR-dependent promoter activity in a *Burkholderia* reporter background [67]. Y2H assays further supported a direct physical interaction between VioS and CviR, consistent with a model in which VioS inhibits CviR activity through PPI [31]. However, as with several LuxR negative regulators discussed above, the precise biochemical mechanism remains unclear and warrants further investigation. Whether VioS interferes with CviR dimerization, DNA binding, or downstream engagement with transcriptional machinery has not been established. Together, these findings establish VioS as an emerging candidate of a negative regulator that directly targets CviR through direct PPI, even in the presence of AHL. Thus, VioS extends the functional spectrum of LuxR negative regulators beyond delayed activation, highlighting the versatility of post-translational control in QS networks.

## Context-dependent regulation of LuxR-type receptors by PPIs

Some LuxR-associated proteins do not function strictly as positive or negative regulators, but instead modulate receptor activity in a context-dependent manner. In these systems, PPIs can enhance or suppress LuxR-dependent transcription depending on promoter architecture, growth phase, or environmental conditions. Rather than acting as general activators, these dual regulators fine-tune QS output by modulating receptor conformation and promoter selection. Below, we highlight representative examples that illustrate how context-dependent PPIs expand the regulatory versatility of LuxR-type receptors. The PqsE-RhlR interaction in *P. aeruginosa* represents the most mechanistically detailed example of this regulatory mode and provides a useful framework for understanding how PPIs can bias LuxR activity in a promoter- and context-dependent manner. By contrast, systems such as the BlsA-AbaR interaction in *Acinetobacter baumannii* represent emerging candidates that suggest similar regulatory principles may operate more broadly, albeit likely through distinct unknown molecular mechanisms.

### PqsE establishes a paradigm as a context-dependent regulator of LuxR-type receptors

The Rhl QS system in *P. aeruginosa* consists of the AHL synthase RhlI, which produces *N*-butanoyl-L-homoserine lactone (C4-HSL), and the cognate receptor RhlR, a LuxR-type transcription factor that activates target genes in response to C4-HSL accumulation [69,70]. RhlR controls the expression of genes encoding a wide array of virulence factors, including rhamnolipids, hydrogen cyanide, and PYO, as well as functions related to biofilm formation and motility [59,70–75]. While RhlR was initially viewed as a canonical AHL-responsive transcriptional activator, it is now evident that its activity is shaped by PPIs that modulate RhlR function in a promoter- and context-dependent manner. Central among these is PqsE, a protein encoded within the *pqs* operon that fine-tunes RhlR-dependent transcription through both positive and negative effects depending on cellular conditions, promoter architecture, and co-factor availability. Rather than acting as a simple activator, PqsE functions as a dual regulator that modulates the amplitude, timing, and specificity of RhlR output.

PqsE was initially characterized as a dispensable thioesterase encoded within the *pqs* operon, involved in quinolone biosynthesis, yet its contribution to QS regulation is mechanistically separable from its enzymatic role [28,76–78]. Deletion of *pqsE* in laboratory strains grown planktonically, resulted in major defects in RhlR-dependent phenotypes, including loss of PYO production, and reduced elastase and hydrogen cyanide synthesis, despite intact C4-HSL and quinolone signaling [79–81]. These phenotypes closely mirror those of *rhlR* mutants and extend to defects in motility, biofilm formation, and *in vivo* pathogenesis [71,78,80]. These observations led to the long-standing hypothesis that PqsE functions as a critical co-regulator to RhlR. Supporting this hypothesis, PqsE enhances RhlR-dependent transcription in heterologous *E. coli* reporter systems lacking other *P. aeruginosa*-specific QS components, and can do so independently of PqsR or PQS production [79]. Together, these observations established PqsE as a critical regulator of RhlR.

Direct biochemical and genetic evidence subsequently demonstrated that PqsE modulates RhlR through a physical PPI. Affinity-purification experiments showed that RhlR co-purifies with PqsE *in vitro* [28], and site-directed mutagenesis subsequently identified a cluster of surface-exposed residues on PqsE critical for binding RhlR. Disruption of this interface abolished PqsE-dependent enhancement of RhlR-DNA binding and transcriptional output, whereas catalytic inactivation of PqsE (PqsE D73A) does not impair RhlR interaction, confirming the regulatory role of PqsE is distinct from its enzymatic activity [28,29]. Notably, a buried mutation within the PqsE active site (PqsE E182W) abolishes RhlR-binding despite being distal to the RhlR interaction interface, suggesting an allosteric coupling between the enzymatic core of PqsE and the RhlR interaction site [28]. While the physiological implications of this allostery remain unclear, it raises the intriguing possibility that conformational states of PqsE, modulated by its active site, could influence its ability to engage RhlR. In support of this regulatory relationship, structural studies showed that PqsE and RhlR form a functional tetrameric complex composed of homodimers of each protein, with PqsE potentially stabilizing a conformation of RhlR that promotes DNA binding at virulence-associated promoters [82,83].

Genome-wide analyses underscore the context-dependent nature of this interaction. Chromatin immunoprecipitation followed by sequencing (ChIP-seq) revealed that PqsE and C4-HSL co-regulate RhlR-DNA binding as well as its ability to activate transcription at 40 genomic loci [84]. These findings demonstrate that PqsE is essential not only for enhancing the occupancy of RhlR at target promoters, but also for driving transcriptional activation of a subset of RhlR-regulated genes. Therefore, RhlR target sites may fall into several categories, some of which require both PqsE and C4-HSL, others depend on only one, and a subset appear independent of both co-factors. These findings indicate that PqsE does not uniformly enhance RhlR activity across the regulon, but instead modulates RhlR function in a promoter-specific manner. This combinatorial regulation suggests that ligand-binding and PqsE interaction cooperatively shape RhlR conformation and transcriptional output.

Physiological context further influences the relative contributions of PqsE and AHL signaling to RhlR-dependent gene expression. During planktonic growth at 37 °C, both PqsE and RhlI-synthesized C4-HSL are required for activation of the *phz* operons and PYO production, consistent with a model in which ligand binding and PqsE cooperatively stabilize a transcriptionally active RhlR complex [84]. In contrast, under surface-associated growth in colony biofilms, *rhlI* mutants retain substantial *phz* transcript accumulation despite the absence of canonical AHL synthesis [80], suggesting that PqsE-dependent regulation of RhlR can partially compensate for reduced AHL availability, or that alternative regulatory inputs converge on RhlR at these promoters. Temperature further influences this balance; RhlR-dependent transcription is elevated at lower temperatures even in the absence of *pqsE*, as observed using chromosomal *phzA* reporters [85], while RhlR protein levels increase at higher temperatures through post-transcriptional thermoregulation of the *rhlAB-rhlR* operon [86]. Together, these findings indicate that the balance between ligand- and PqsE-mediated control of RhlR is highly context-dependent and shaped by growth phase and environmental conditions.

Consistent with this view, recent studies using well-defined PqsE variants with altered RhlR-binding affinities revealed that PqsE can exert both positive and negative effects on gene expression depending on the target promoter [87]. These findings suggest that RhlR may adopt multiple active conformations, and PqsE stabilizes one such conformation, potentially the only form capable of activating certain promoters, but suboptimal or even inhibitory at others. This repressive function appears to be biologically relevant; elevated PqsE levels suppress expression of a subset of RhlR-regulated genes, including *vqsR* and *clpP2*, demonstrating that PqsE fine-tunes the RhlR regulon, rather than acting as a general amplifier of expression [88]. The regulatory complexity of the PqsE-RhlR interaction is further underscored by reciprocal feedback loops and post-translational regulation. RhlR represses *pqsE* expression through the *pqsA* promoter, with repression enhanced when the PqsE-RhlR complex is intact [79,89]. This creates a negative feedback loop: increased RhlR activity suppresses *pqsE* expression, thereby limiting further PqsE-mediated enhancement of RhlR [90,91]. Conversely, when RhlR or C4-HSL levels decline, *pqsE* expression increases, potentially reinforcing RhlR-dependent transcription at select PqsE-dependent promoters [87]. In support of this model, overexpression of *pqsE* leads to increased RhlR protein abundance without corresponding changes to *rhlR* transcripts or mRNA translation, suggesting a post-translational mechanism [92]. Separately, PqsE has been proposed to shield RhlR from proteolysis (discussed further below), suggesting a stabilizing role that may help preserve receptor abundance during QS activation [93]. This stabilizing function requires PqsE homodimerization; monomeric variants fail to bind RhlR or enhance its activity, demonstrating that PqsE dimerization is essential for complex assembly and RhlR modulation [29,93]. Given the negative feedback between PqsE and RhlR, it is likely that PqsE dimerization itself is under homeostatic regulation, serving as a point of QS-dependent control. This requirement for dimerization may serve as a regulatory checkpoint, ensuring that RhlR is stabilized and fully active only when PqsE accumulates and dimerizes, thereby restricting RhlR-dependent gene expression of virulence factors to conditions when QS thresholds have been robustly met.

Evolutionary analyses showed that PqsE is highly conserved across *P. aeruginosa* isolates, with strict amino acid identity at residues essential for PqsE homodimerization and RhlR-binding [93]. Importantly, while there are a limited number of species that encode a *pqsE* homolog, the PqsE-RhlR interaction has been observed in closely related species [92,93]. However, recent work examining PqsE from diverse *P. aeruginosa* isolates revealed that, despite strong sequence conservation, the contribution of PqsE to QS-regulated phenotypes varies substantially between strains. Trottier *et al*. showed that deletion of *pqsE* produces strain-dependent effects on virulence outputs, indicating that PqsE function is modulated by genetic background and regulatory context rather than being universally interchangeable across strains [94]. These findings underscore that PqsE activity cannot be inferred from sequence conservation alone and further support a model in which PqsE acts as a context-dependent modulator of RhlR-mediated transcription. In contrast, orthologs from *Burkholderia* species lack the structural features required for homodimerization or LuxR receptor interaction, supporting the model that this PPI evolved specifically to modulate QS in *P. aeruginosa* [93]. Importantly, many virulence genes in *P. aeruginosa* are co-regulated by both PqsE and RhlR, which makes both components indispensable for full pathogenesis. Disruption of the PqsE-RhlR interface abolishes PYO production and impairs colonization of the murine lung [71]. Accordingly, inhibitors targeting either RhlR or PqsE downregulate biofilm formation and virulence factor expression, highlighting this PPI as a promising target for anti-virulence therapies [95–99]. Together, these findings establish PqsE as a paradigm for context-dependent modulation of LuxR-type receptor activity through PPI.

### BlsA-mediated context-dependent regulation of AbaR in *A. baumannii*

A parallel example of context-dependent LuxR modulation is found in *A. baumannii*, an opportunistic pathogen with a QS system composed of the AHL synthase AbaI, and the LuxR‑type receptor AbaR [100–103]. While AbaR was initially considered a canonical AHL-responsive LuxR-type receptor, subsequent work revealed an additional regulatory layer mediated by the blue light-sensing protein, BlsA. BlsA expression is highest at lower temperatures in the dark; under these conditions, BlsA interacts with multiple protein partners, including AbaR [27,104,105]. The BlsA-AbaR interaction is proposed to induce expression of the AHL synthase *abaI* and the subsequent production of AHL. By contrast, under blue light, BlsA does not interact with AbaR and instead induces expression of *aidA*, encoding a quorum-quenching lactonase, resulting in reduced QS output [27]. While these studies collectively support a model in which BlsA acts as a context-dependent modulator whose expression and interactions are tuned by environmental conditions, the precise mechanism remains unknown. Future work will be critical to determine how BlsA modulates AbaR activity at the molecular level, whether by stabilizing protein conformation, facilitating dimerization, or altering ligand responsiveness, and to establish whether BlsA is required for full AbaR function *in vivo*. Clarifying this mechanism will not only advance our understanding of QS regulation in *A. baumannii* but may also uncover new targets for anti-virulence therapeutics should the interaction increase virulence.

**Fig 5:**
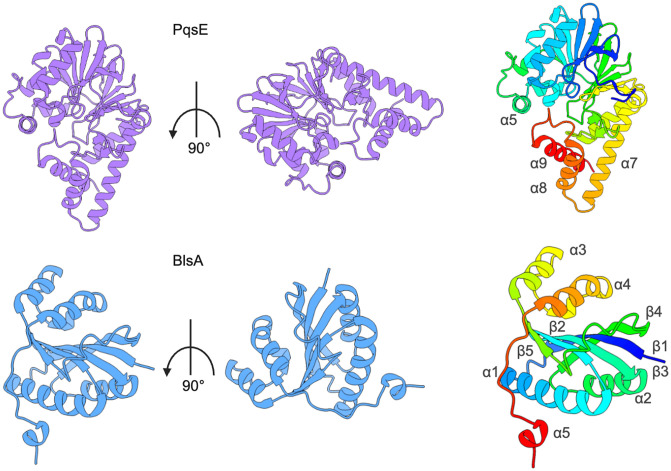
Structural comparison of LuxR-type context-dependent regulators. **(A)** Cartoon representation of a PqsE monomer (purple; PDB ID: 7KGW [28]) rotated 90° on its axis and colored by rainbow from the N-terminus to the C-terminus. Highlighted are conserved C-terminal alpha helices, α5–α9, that are unique among this class of enzymes. **(B)** Cartoon representation of BlsA (blue; PDB ID: 6W6Z [105]) in the ground state, rotated by 90° on its axis and colored by rainbow from the N-terminus to the C-terminus. Highlighted is the secondary structure of BlsA, which is notably different compared to PqsE. All structure images were generated using ChimeraX.

Interestingly, while AbaR shares modest sequence homology with RhlR, the accessory proteins BlsA and PqsE are structurally and evolutionarily unrelated (Fig 5) [93]. Despite this, both proteins modulate LuxR-type receptor activity through direct interaction, highlighting a broader evolutionary trend in which proteins with enzymatic functions appear to have evolved separate, non-enzymatic roles to regulate LuxR-type receptor activity. These findings highlight the remarkable versatility of such proteins in expanding the regulatory capacity of QS in response to environmental cues.

## Global regulators

In *Vibrio* species, LuxR (TetR-family proteins) receptors serve as canonical transcriptional activators that regulate gene expression through direct engagement of RNA polymerase (RNAP). Extensive genetic and biochemical studies in these systems have established that LuxR receptors activate transcription by binding promoter-proximal sites and facilitating RNAP recruitment, often through contacts with the C-terminal domain of the RNAP α subunit (α-CTD) [106]. This mechanism provides the prevailing paradigm for how LuxR-type receptors are thought to function across Gram-negative bacteria.

**RNA polymerase** **engagement**

**Fig 6:**
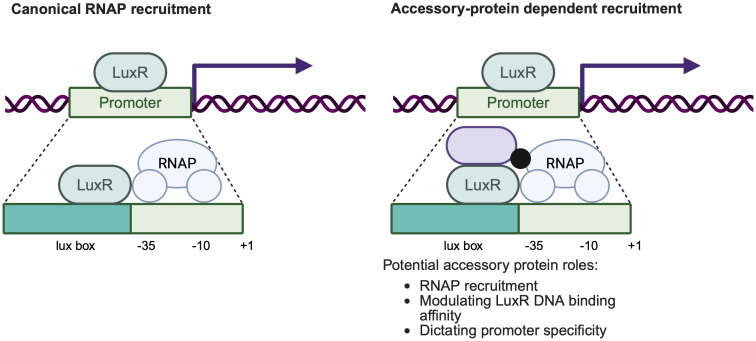
Canonical and accessory protein dependent models of LuxR-RNAP recruitment. Canonical LuxR proteins recruit RNAP directly when bound to promoter-proximal lux boxes, typically upstream of the -35 element (left), as established in *Vibrio* species. In contrast, some LuxR-type receptors may require accessory proteins to enable transcription potentially through RNAP recruitment, modulation of DNA binding, or promoter specificity (right). This accessory-mediated mechanism remains hypothetical and likely reflects a layer of context-dependent regulation. Created in BioRender. Paczkowski, J. (2026) https://BioRender.com/kv3bnqa.

Multiple independent lines of evidence from *Vibrio* and *Agrobacterium* systems support direct functional engagement between LuxR receptors and RNAP. LuxR and TraR mutants have been identified that retain DNA binding but fail to activate transcription and instead act as repressors, consistent with defects in RNAP engagement rather than promoter recognition [107,108]. Complementary genetic studies identified RNAP α-subunit mutants that are specifically impaired in LuxR- or TraR-dependent activation while leaving basal transcription intact, indicating that productive transcription requires synergistic interactions between LuxR-type receptors and RNAP [106,109]. Promoter architecture further supports this model: the lux box is positioned immediately upstream of the −35 RNAP binding element, and repositioning this site eliminated transcriptional activation (Fig 6) [110]. Notably, neither LuxR nor RNAP binds efficiently to target promoters alone, whereas together they form stable, transcriptionally competent complexes [111]. Collectively, these observations strongly imply direct physical interaction between LuxR receptors and RNAP, even in the absence of formal PPI assays.

To date, CviR from *C. violaceum* ATCC 12472 remains the only AHL-binding LuxR-type receptor for which a direct PPI with RNAP has been demonstrated using interaction assays [112]. Using BACTH analysis, CviR bound to its cognate ligand C6-HSL was shown to interact with the C-terminal domain of the RNAP α-subunit (α-CTD), whereas antagonist-bound or non-cognate ligand-bound forms failed to do so. This ligand-dependent interaction correlates with structural models in which C6-HSL binding stabilizes an “open” CviR conformation compatible with transcriptional activation, while antagonist-bound forms adopt a closed, repressive configuration [112]. These findings provide direct evidence that LuxR-type receptors can recruit RNAP in a ligand-dependent manner.

Recent work in *P. aeruginosa* suggests that RNAP engagement may represent a key regulatory checkpoint modulated by PPIs. The LuxR-type receptor RhlR can occupy target promoters in the absence of the accessory protein PqsE, yet fails to drive transcription under these conditions. Coupled ChIP-seq and RNA-seq analyses indicate that PqsE is required for transcriptional activation at a subset of RhlR-bound promoters, despite not being strictly required for DNA binding [84]. This uncoupling of promoter occupancy from transcriptional output suggests that PqsE may influence steps downstream of DNA binding, such as stabilizing a transcriptionally competent RhlR conformation or facilitating productive RNAP engagement (Fig 6). Together, these examples support a model in which LuxR-type receptors operate within a conserved transcriptional framework defined by RNAP recruitment, while accessory proteins modulate the efficiency, timing, and promoter specificity of this process. Although LuxR-RNAP engagement is well supported by functional and genetic evidence, the molecular interfaces involved and the extent to which PPIs modulate this step remain largely unexplored. Defining how accessory proteins influence RNAP recruitment represents an important open question in understanding QS activation.

### Proteolytic regulation of LuxR-type receptors

AAA+ proteases are ATP-dependent machines that control protein homeostasis in bacteria by unfolding and degrading targeted substrates. Members of this family, including ClpP, Lon, FtsH, and HslUV, play essential roles in degrading misfolded proteins, resolving toxic aggregates, and regulating key cellular processes through selective proteolysis [113,114]. Beyond general protein quality control, many AAA+ proteases have evolved to act as post-translational regulators of gene expression, by selectively degrading transcription factors in response to environmental or metabolic cues [113,114]. In diverse bacterial systems, transcription factor turnover by AAA+ proteases enables rapid reprogramming of gene expression. In *E. coli*, for example, Lon protease plays a key role in regulating the oxidative stress-response. Under stress conditions, the transcription factor SoxR is activated and induces expression of SoxS, which then activates a set of genes that help the cell detoxify reactive oxygen species [115,116]. Once the stress has passed, Lon rapidly degrades SoxS to shut down the response due to decreased *soxS* transcription, ensuring that stress-response genes are not expressed unnecessarily. This tightly controlled degradation underscores how AAA+ proteases can shape transcriptional programs by degrading unstable regulatory proteins. In *Caulobacter crescentus*, Lon controls the abundance of the DNA replication initiator DnaA [117], and the global transcription factor CtrA, coordinating cell cycle progression with environmental conditions [118]. These and other examples highlight the broad utility of AAA+ proteases in shaping transcriptional networks through the targeted degradation of regulatory proteins.

LuxR-type QS receptors may be particularly well-suited to this form of regulation. These proteins are widely understood to require their cognate AHL ligands not only for transcriptional activation but also for proper folding and protein stability [11,119]. In the absence of ligand, LuxR-type receptors often misfold, fail to dimerize, or adopt nonfunctional conformations, rendering them susceptible to proteolytic degradation [119]. This ligand-dependent folding behavior is thought to serve as a critical physiological checkpoint by limiting receptor accumulation at LCD, when AHL concentrations are insufficient to support QS activation. In doing so, bacteria avoid premature activation of QS-regulated genes. However, whether this inherent instability is actively exploited by proteolytic systems such as Lon or ClpP to shape QS output remains poorly understood. Exploring the post-translational control of LuxR-type receptors represents a promising direction for uncovering new regulatory layers in QS. The clearest example of this principle comes from the LuxR-type receptor TraR in *A. tumefaciens*. Apo-TraR, in the absence of its ligand 3OC8-HSL, is rapidly degraded by Lon and ClpP proteases, with a half-life of 2–3 min, and cannot fold into its functional dimeric form [119]. Only TraR synthesized in the presence of 3OC8-HSL adopts a folded, dimeric, and protease-resistant conformation. Follow-up work by Costa *et al*. expanded this model by identifying intrinsic degradation signals within the C-terminal DBD of TraR, including a protease recognition motif near residues 184–187 [120]. Mutation of these residues increased TraR half-life, while fusion of this domain to GFP reduced fluorescence, suggesting that these C-terminal residues function as a degron to ensure protein turnover. Moreover, this study revealed that the anti-activator TraM acts as a proteolytic adaptor, forming 2:2 complexes with TraR that prevent DNA binding and direct TraR for degradation. Importantly, evidence for TraM-dependent TraR turnover derives primarily from cell-based expression assays in which TraM levels were modulated by IPTG induction. Increasing TraM expression resulted in decreased TraR abundance, as assessed by immunoblotting; however, these experiments did not include quantitative loading controls, and altered TraR levels could also reflect indirect effects of TraM overexpression on TraR synthesis or cellular resource allocation. Notably, TraM itself is not degraded in this process and may act catalytically to promote turnover of multiple TraR molecules [120]. Therefore, while the data strongly support a model in which TraM antagonizes TraR activity and promotes its degradation, whether TraM directly stimulates proteolysis or indirectly destabilizes TraR remains to be fully resolved. Together, these findings establish TraM as a potent negative regulator of TraR and highlight regulated proteolysis as an important, though still incompletely defined, layer of LuxR-type receptor control. Together, these studies illustrate how proteolysis and ligand-dependent folding intersect to tightly regulate LuxR-type receptor abundance, and how PPIs (*e.g*., with TraM) modulate both activity and stability. These findings highlight proteolysis as a key regulatory layer in QS, particularly in resetting QS systems when signal levels decline. This may be a broadly conserved mechanism among LuxR-type homologs with unstable apo-forms. This “fold-to-function” model has become a conceptual framework for understanding other LuxR-type regulators.

In *P. aeruginosa*, early work by Takaya *et al*. investigated whether the ATP-dependent Lon protease plays a role in regulating LuxR-type receptors [121]. They observed that Δ*lon* mutants accumulated elevated levels of RhlR and overproduced QS-regulated virulence factors such as PYO. However, pulse-chase labeling indicated that RhlR had a similar half-life in both wild-type and Δ*lon* backgrounds. Furthermore, increased *rhlR* mRNA levels in the *lon* mutant pointed to indirect effects [121]. These findings led to the conclusion that Lon does not degrade RhlR directly, but instead modulates QS indirectly through degradation of the AHL synthase LasI and effects on upstream regulators like RhlI. Indeed, subsequent studies demonstrated that AHL synthases such as LasI [121] and RhlI [122–124] are direct Lon and ClpXP substrates, revealing that proteolysis plays a broader role in QS by modulating signal production and upstream regulators.

Recent findings have added new complexity by identifying a condition under which RhlR becomes a direct Lon substrate: in the absence of the accessory protein PqsE [93]. In PA14, genetic backgrounds lacking *pqsE* or the ability of PqsE to dimerize and interact with RhlR, showed significantly reduced RhlR protein levels. This reduction was rescued in Δ*pqsE*Δ*lon* double mutants, indicating that RhlR is targeted by Lon in the absence of *pqsE* [93]. Importantly, the partial rescue (to WT rather than Δ*lon* levels) suggests that additional proteases may also contribute to RhlR turnover. Notably, these experiments were performed in PA14, whereas earlier studies, including those by Takaya *et al*., used PAO1 [93,121]. This distinction is important, as regulatory differences between PAO1 and PA14 are well documented and may influence protease-substrate interactions [62]. These findings support a model in which PqsE stabilizes RhlR by preventing its recognition by Lon and possibly other proteases, likely through concentration-dependent interactions. However, this model remains to be fully validated, and future work will be essential to define the complete set of proteases involved, their substrate preferences, and how receptor conformation or accessory protein-binding modulates proteolytic susceptibility.

Despite these advances, many key questions remain unanswered. What structural features of LuxR-type receptors mark them for degradation? Which additional proteases, beyond Lon and ClpXP, target QS components in *P. aeruginosa* under various physiological conditions? How do dynamic changes in ligand occupancy or accessory protein-binding influence susceptibility to proteolysis? Future work combining genetic and biochemical studies will be essential to delineate the full proteolytic network that governs LuxR-type receptor stability.

## Outlooks and perspectives

The discovery of direct PPIs that regulate LuxR-type receptors has significantly expanded our understanding of QS regulation. These interactions reveal that LuxR-type proteins are not merely ligand-activated transcription factors that can switch on or off gene expression; instead, they are subject to an evolving network of regulators that fine-tune their stability, activity, and specificity. Across Gram-negative bacteria, diverse accessory proteins, ranging from biosynthetic enzymes like PqsE to light sensors like BlsA, have been co-opted to modulate LuxR-type receptor function through non-enzymatic interactions. Some PPI stabilize active receptor conformations, while others act as anti-activators or direct repressors, preventing premature or inappropriate QS activation. This layered control enables bacterial populations to balance collective behavior with species-specific responsiveness, particularly in fluctuating or heterogeneous environments. Despite the growing list of LuxR-interacting proteins, many key questions remain. What structural features dictate binding specificity between LuxR-type receptors and their partners? Are anti-activators like QteE and TraM functionally conserved across species or examples of convergent evolution? Do all LuxR-type receptors interface with proteostasis networks, or is this limited to unstable, ligand-dependent forms? Moreover, how do environmental inputs, such as light, host-derived molecules, or nutrient status, influence the formation of LuxR-protein complexes?

From a therapeutic standpoint, targeting LuxR-interacting proteins may offer a promising alternative to conventional QS disruption strategies. Inhibiting key interactions, such as the PqsE-RhlR complex, has already been shown to attenuate virulence in a murine infection model without impacting bacterial growth [37]. Understanding the structural and regulatory mechanisms of these interactions could pave the way for species-specific anti-virulence therapies that avoid broad-spectrum resistance pressures. In the broader context of microbial regulation, LuxR PPIs exemplify a general theme: transcription factors often function within protein complexes that integrate multiple signals and post-translational inputs. Continued study of LuxR-protein interactions, spanning genetics, structural biology, and comparative genomics, will be essential for uncovering new principles of bacterial communication, adaptability, and pathogenesis.
